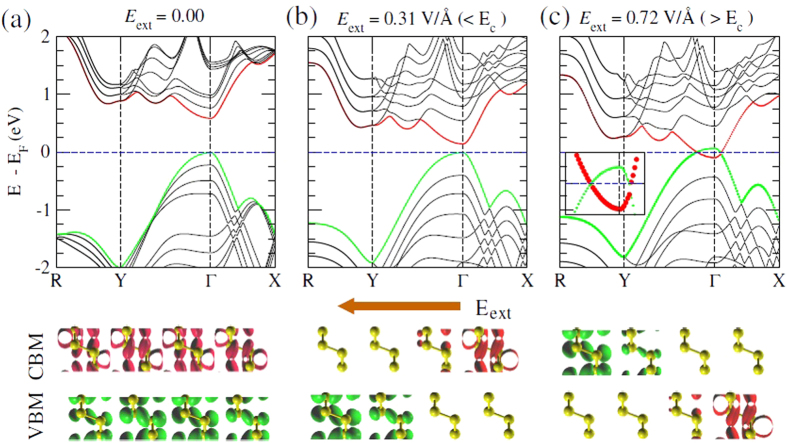# Corrigendum: Quantum-confinement and Structural Anisotropy result in Electrically-Tunable Dirac Cone in Few-layer Black Phosphorous

**DOI:** 10.1038/srep25429

**Published:** 2016-05-09

**Authors:** Kapildeb Dolui, Su Ying Quek

Scientific Reports 5: Article number: 11699;10.1038/srep11699 Published online: 07012015; Updated: 05092016

This Article contains an error in Figure 2: the arrow depicting the electric field should point right to left. The correct Figure 2 appears below as Figure 1.

## Figures and Tables

**Figure 1:**