# Supplementary material for: Quantum-confinement and Structural Anisotropy result in Electrically-Tunable Dirac Cone in Few-layer Black Phosphorous
Source: Sci Rep. 2015 Jul 1;5:11699. doi: 10.1038/srep11699 (PMC4486999; doi:10.1038/srep11699)
Supplement: Supplementary Information [file srep11699-s1.pdf]

Supplementary Information for "Quantum-confinement and Structural Anisotropy result in Electrically-Tunable Dirac Cone in Few-layer Black Phosphorous"

Kapildeb Dolui[1] and Su Ying Quek[1,2] *

[1] *Department of Physics, Centre for Advanced 2D Materials and Graphene Research Centre, Faculty of Science, National University of Singapore, 2 Science Drive 3, Singapore 117551*

[1,2] *Institute of High Performance Computing, 1 Fusionopolis Way, # 16-16 Connexis, Singapore 138632 and*

**Corresponding author: phyqsy@nus.edu.sg*

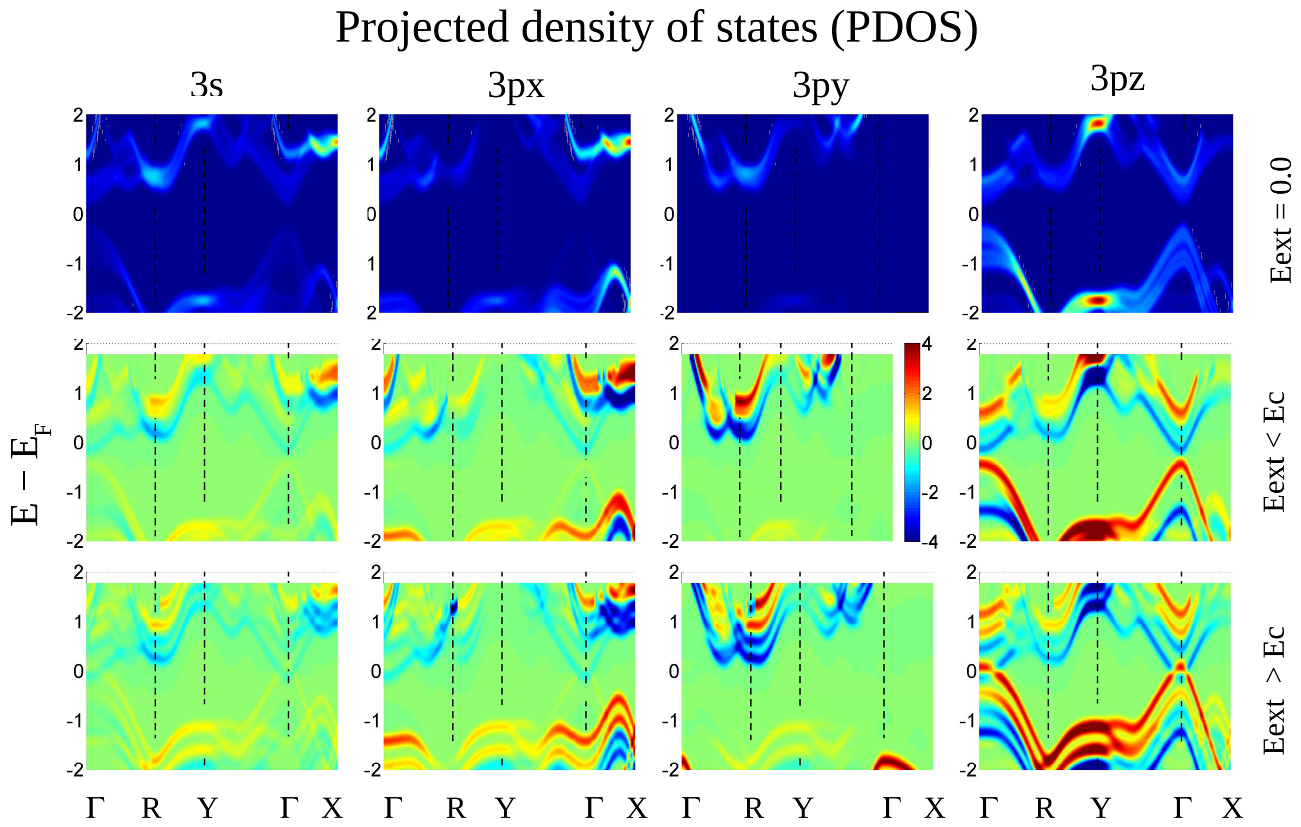

FIG. 1: **Supporting Figure S1:** The band-decomposed projected density of states (PDOS) on different orbitals for 4 layer black phosphorus. The densities of states are projected on $3s$, $3p_x$, $3p_y$ and $3p_z$ orbitals. Upper, middle and lower panels show the PDOS at $E_{\rm ext}$ = 0.00, 0.21, and 0.72 V/Å, respectively. Note that the critical field is E_c = 0.48 V/Å. At $E_{\rm ext}$ = 0, the PDOS is summed over all the atoms in the unit-cell. For finite fields, for visualization purposes, we use the negative colour scale for PDOS that comes from the two layers kept at positive potential, and the positive colour scale for PDOS coming from the other two layers kept at negative potential, as indicated in Figure 1 in the main text.

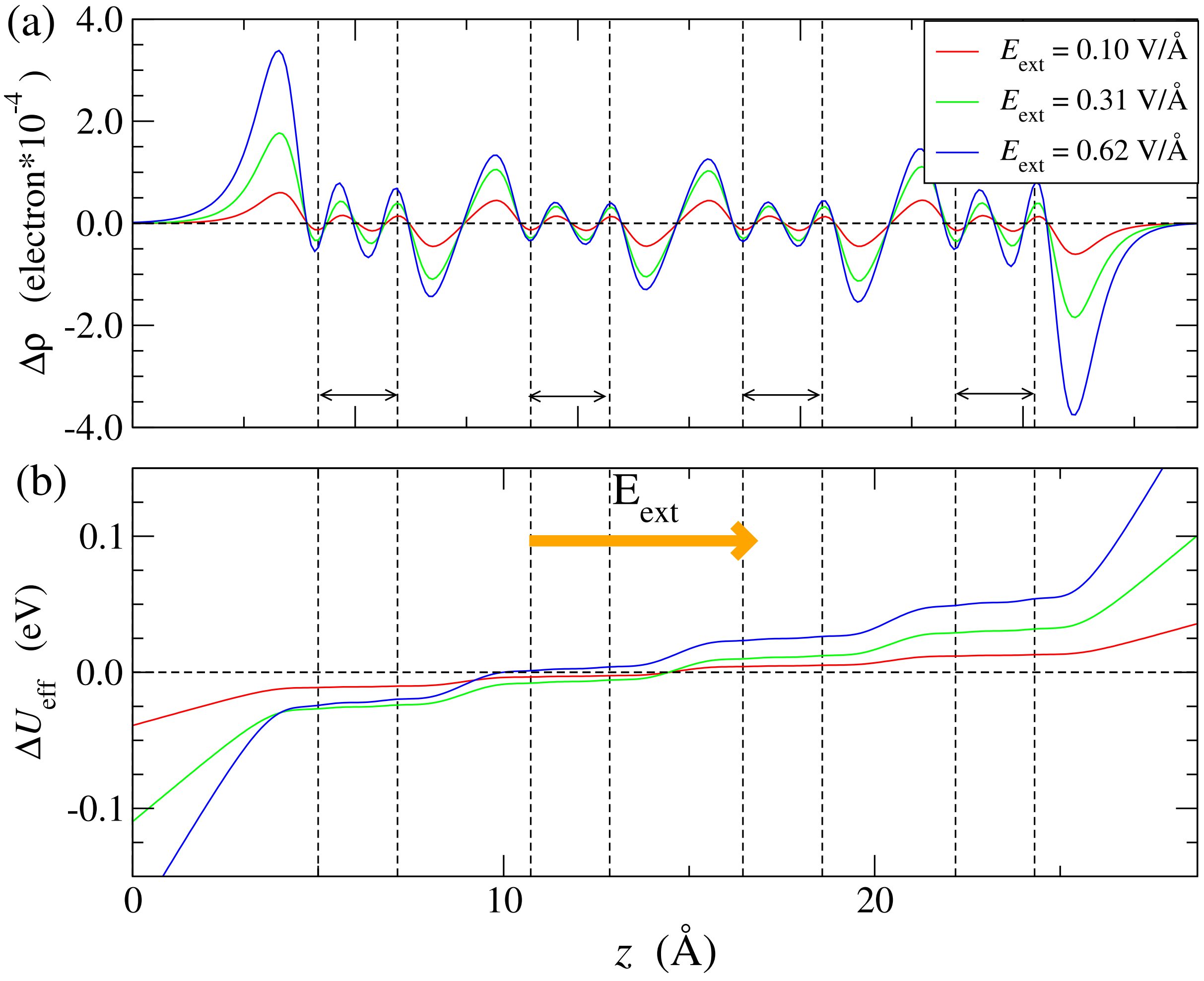

FIG. 2: **Supporting Figure S2:** (a) Electric field induced charge density distribution across the layers, $\Delta\rho = \rho(E_{\rm ext}) - \rho(E_{\rm ext} = 0)$, for 4L-BP and different values of $E_{\rm ext}$. $\rho(E_{\rm ext})$ is the charge density for an external field, $E_{\rm ext}$. $\rho(E_{\rm ext})$ is averaged over the transverse direction (xy-plane) and plotted along the out-of-plane direction (z-axis). The dashed vertical lines denote the positions of each atomic plane in 4L-BP. (b) Effective electrostatic potential across the layers, $\Delta U_{\rm eff} = U(E_{\rm ext}) - U(E_{\rm ext} = 0)$, for 4L-BP and different values of $E_{\rm ext}$.

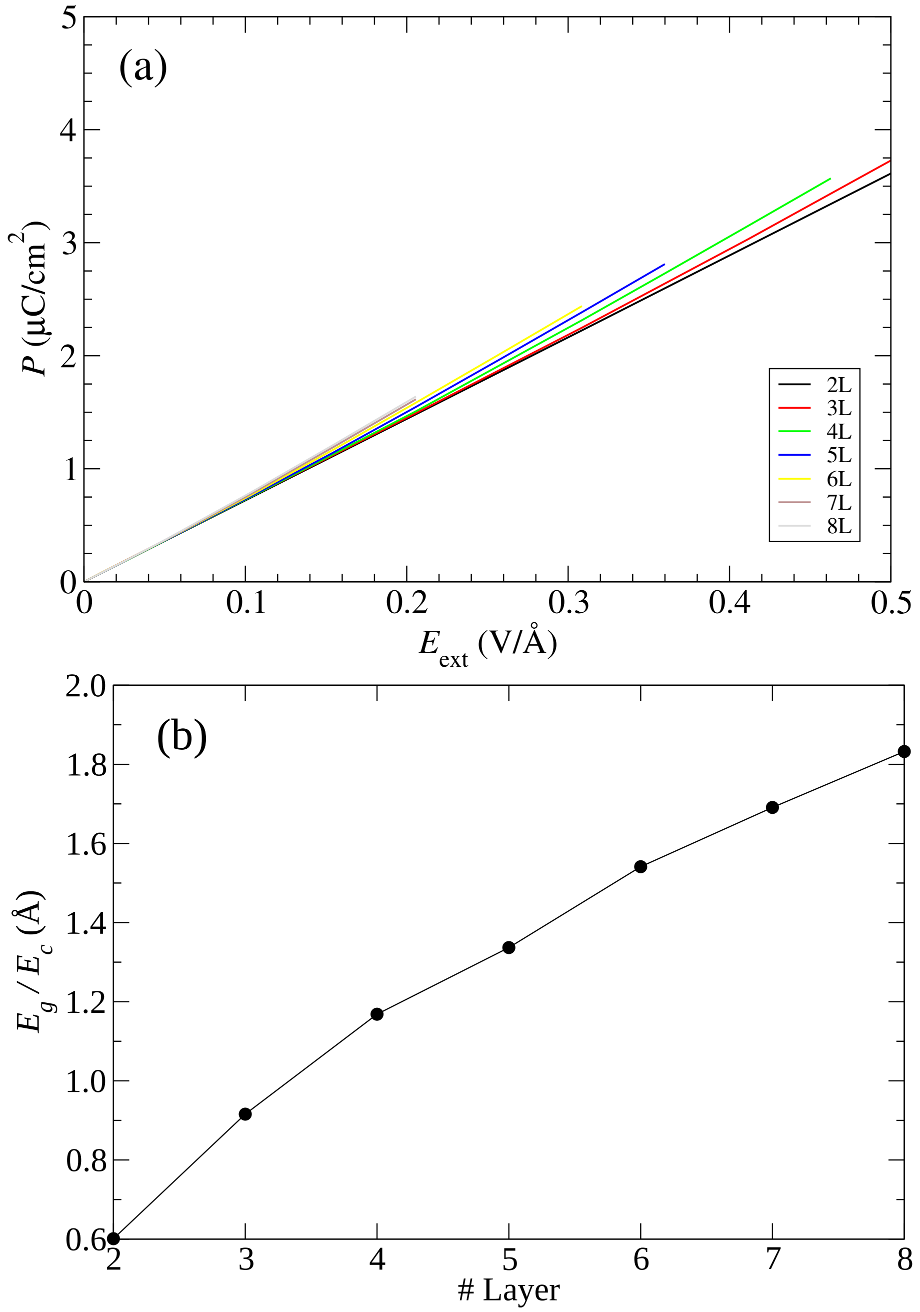

FIG. 3: **Supporting Figure S3:** (a) The polarization (dipole moment per unit area) , P, is plotted against the applied external field strength for 2L (black), 3L (red), 4L (green), 5L (blue), 6L (yellow), 7L (brown) and 8L (grey)-BP. (b) (E_g/E_c is plotted as a function of the number of layers.

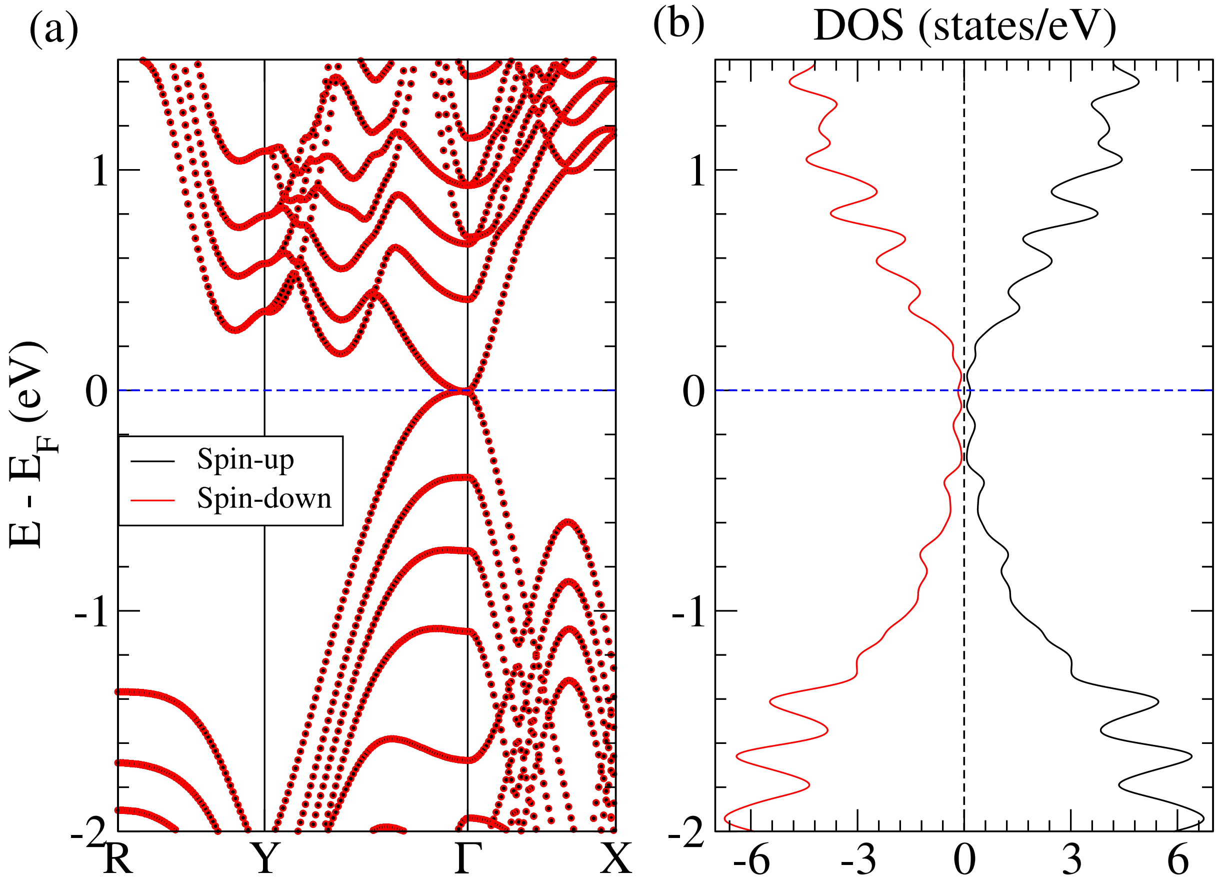

FIG. 4: **Supporting Figure S4:** (a) Spin-polarized bandstructure and (b) density of states of 4L-BP. The blue dashed line denotes the Fermi level. Red and black lines denote the spin-up and spin-down component of the DOS, respectively.

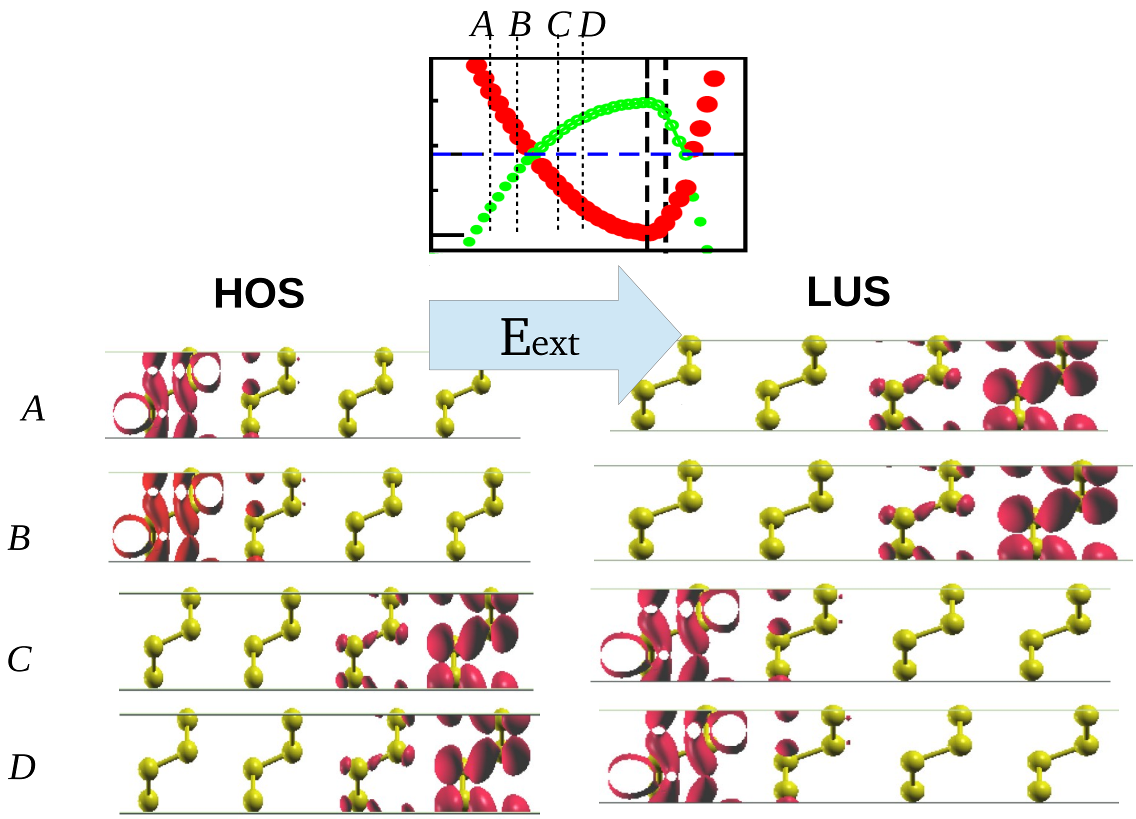

FIG. 5: **Supporting Figure S5:** The local density of states (LDOS) of highest occupied states (HOS) left) and lowest unoccupied states (LUS) (right) at the different k-points around the Dirac point, Λ, as indicated in the bandstucture (top). The figure shows that band inversion occurs at the Λ point.

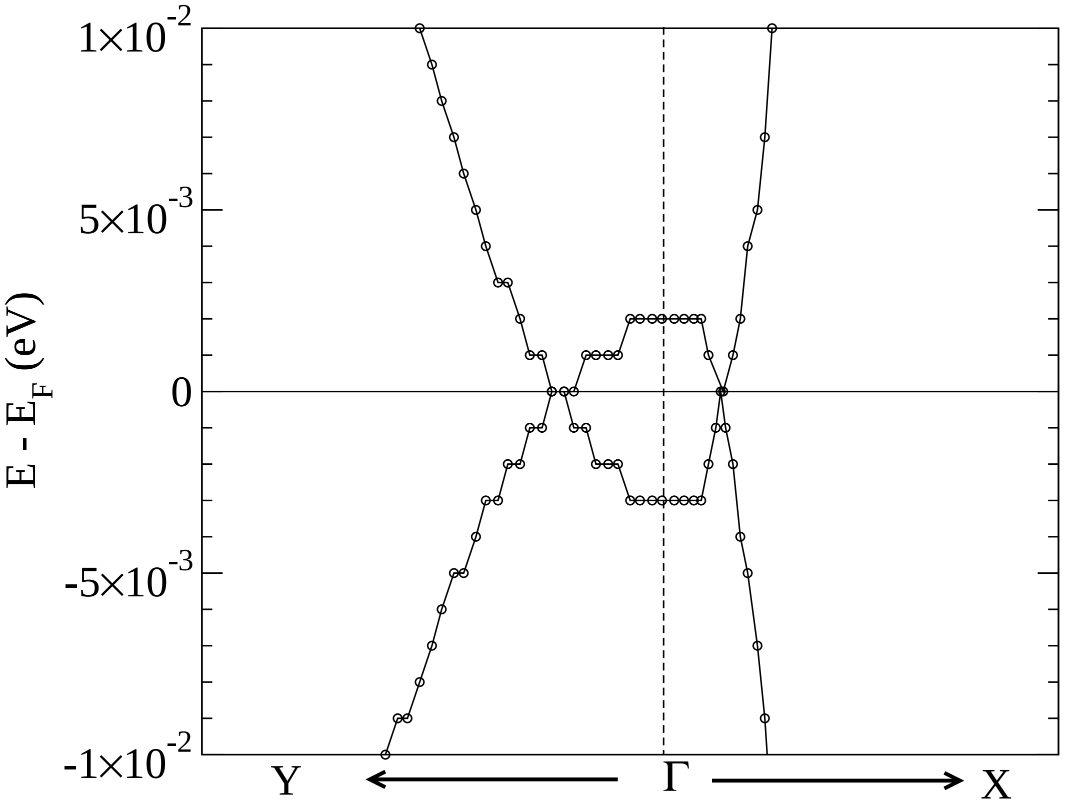

FIG. 6: **Supporting Figure S6:** The bandstructure of 12L-BP above the critical field, i.e., E_{ext}= 0.26 V/Å.

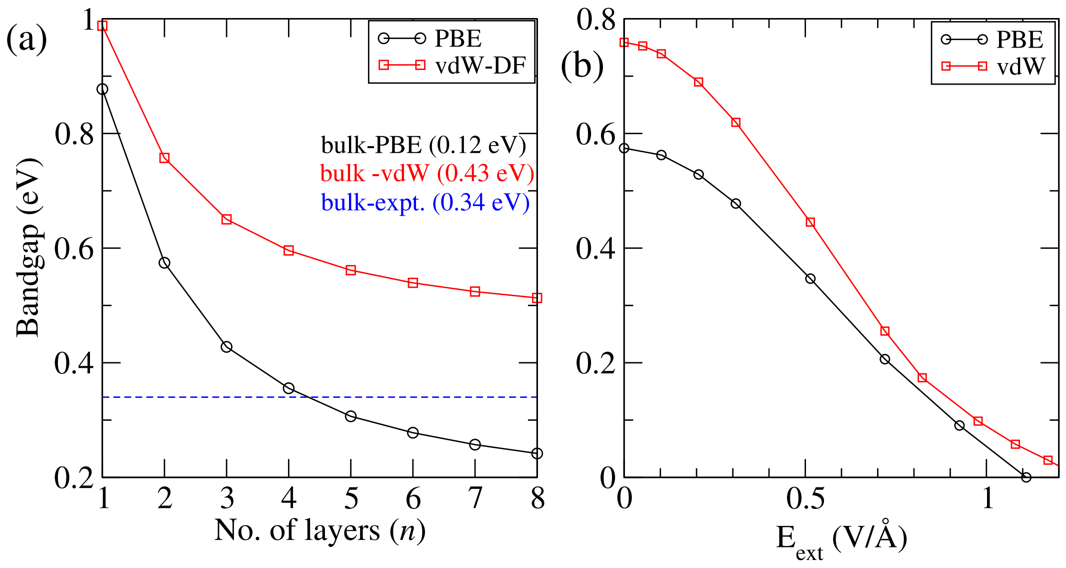

FIG. 7: **Supporting Figure S7:** Comparison of electronic structure for different functionals. (a) The variation of the band-gaps calculated within PBE(black line) and vdW-corrected(red line) exchange correlation functionals, plotted as a function of the number of layers. Note that the PBE band gap of bulk BP is 0.12 eV, while the vdW-corrected band gap is 0.34 eV. The dashed line denotes the value of the experimental band gap (0.34 eV) of bulk BP. (b) The variation of the band gap of bilayer BP, calculated within PBE(black line) and vdW-corrected(red line) functionals, plotted against the applied field strength.

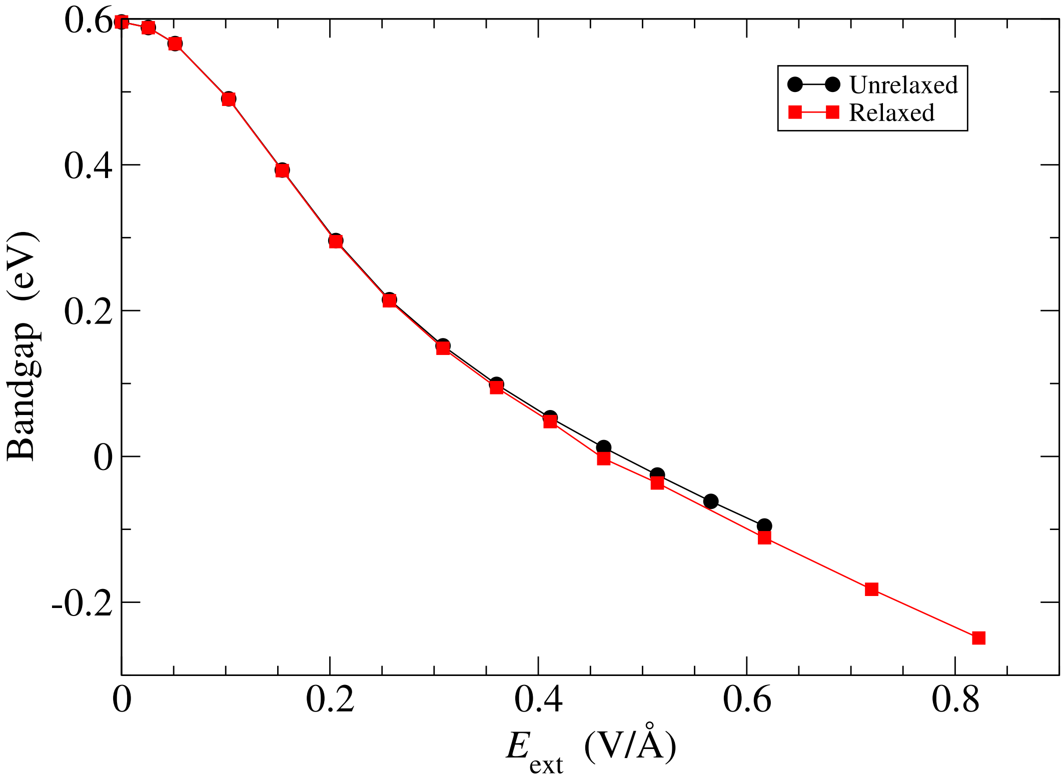

FIG. 8: **Supporting Figure S8:** The bandgap of 4L-BP as a functional of E_{ext} using the unrelaxed (black) and relaxed (red) coordinates under an applied field E_{ext}.
